# Treating Extensive Spontaneous Osteonecrosis of the Tibia by the Medial Opening Wedge High Tibial Osteotomy Procedure: A Case Report

**DOI:** 10.7759/cureus.67336

**Published:** 2024-08-20

**Authors:** Yuzuru Sakakibara, Rikiya Mukai, Kohei Shimoyama, Yohei Okada, Atsushi Teramoto

**Affiliations:** 1 Orthopaedic Surgery, Muroran City General Hospital, Sapporo, JPN; 2 Orthopaedic Surgery, Sapporo Medical University School of Medicine, Sapporo, JPN

**Keywords:** magnetic resonance imaging, medial opening wedge osteotomy, high tibial osteotomy, spontaneous osteonecrosis, arthroscopy

## Abstract

Osteonecrosis of the tibia is less common than that of the femoral condyle, with no consensus on surgical indications. In this study, a medial opening wedge high tibial osteotomy (OWHTO) was performed to treat the very extensive osteonecrosis of the tibia. This case demonstrates significant symptomatic relief and functional improvement following OWHTO for spontaneous tibial plateau osteonecrosis. The findings support the hypothesis that changes in mechanical stress contribute to disease progression. The promising results of this case study highlight the need for further studies to confirm its efficacy in a larger patient cohort, sparking interest in the future of this field. This case report is complemented by a literature review, which provides insights into management based on the patient's clinical course.

## Introduction

Osteonecrosis of the tibia, a condition characterized by necrosis of bone tissue due to a lack of blood supply, significantly affects patient activity and quality of life. Although affecting a broad demographic population, its prevalence is notably higher among individuals aged 60 years and older, with spontaneous cases often leading to severe degenerative changes in the knee joint [1-4]. The pathophysiology of spontaneous osteonecrosis remains complex and involves factors such as microvascular thrombosis and increased intraosseous pressure, ultimately resulting in the collapse of the tibial plateau and chronic knee pain [5,6]. Historically, the surgical treatment landscape for osteonecrosis has evolved from total knee arthroplasty (TKA) to more limb-preserving procedures, such as high tibial osteotomy (HTO) that aims to redistribute joint loading away from the affected area. Medial opening wedge high tibial osteotomy (OWHTO), in particular, has gained prominence because of its ability to correct varus malalignment and improve joint congruence without the need for knee joint replacement [7-10]. Comparative studies have demonstrated that patients undergoing HTO for knee pathologies exhibit significant improvement in pain and function, with success rates varying between 70% and 90% over 5-10 years post-operation [7,11]. The objective of this case report was to detail the application of medial OWHTO in treating a patient with extensive spontaneous osteonecrosis of the tibia, providing much-needed insight into this specific intervention.

## Case presentation

**Figure 1:**
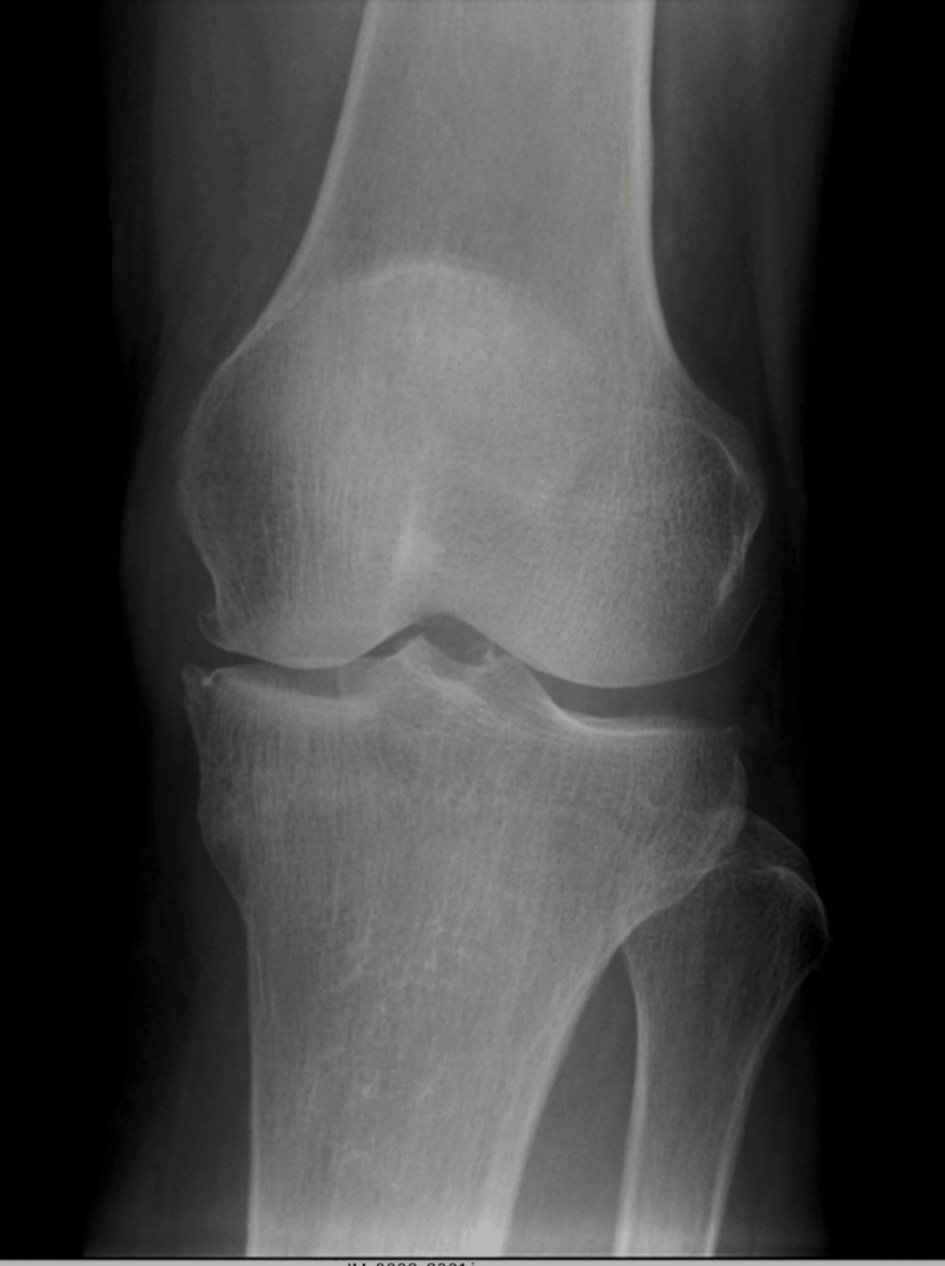
An X-ray at the time of initial examination. An X-ray at the time of initial examination showed a medial joint space narrowing and bony spur.

A 61-year-old woman presented with left knee pain. Alcohol intake was around 1.1 units per day. There was no history of steroid use. The current medical history of the patient included experiencing left knee pain for two years without any particular trigger. The patient visited our clinic a few weeks prior when she noticed a sharp increase in her knee pain and started having trouble walking. She had no spontaneous pain and nocturnal pain but had prominent pain during loading and walking. Medial joint space tenderness was also noted. Radiographs showed a narrowing of the medial joint space and the presence of osteophytes (Figure 1).

**Figure 2:**
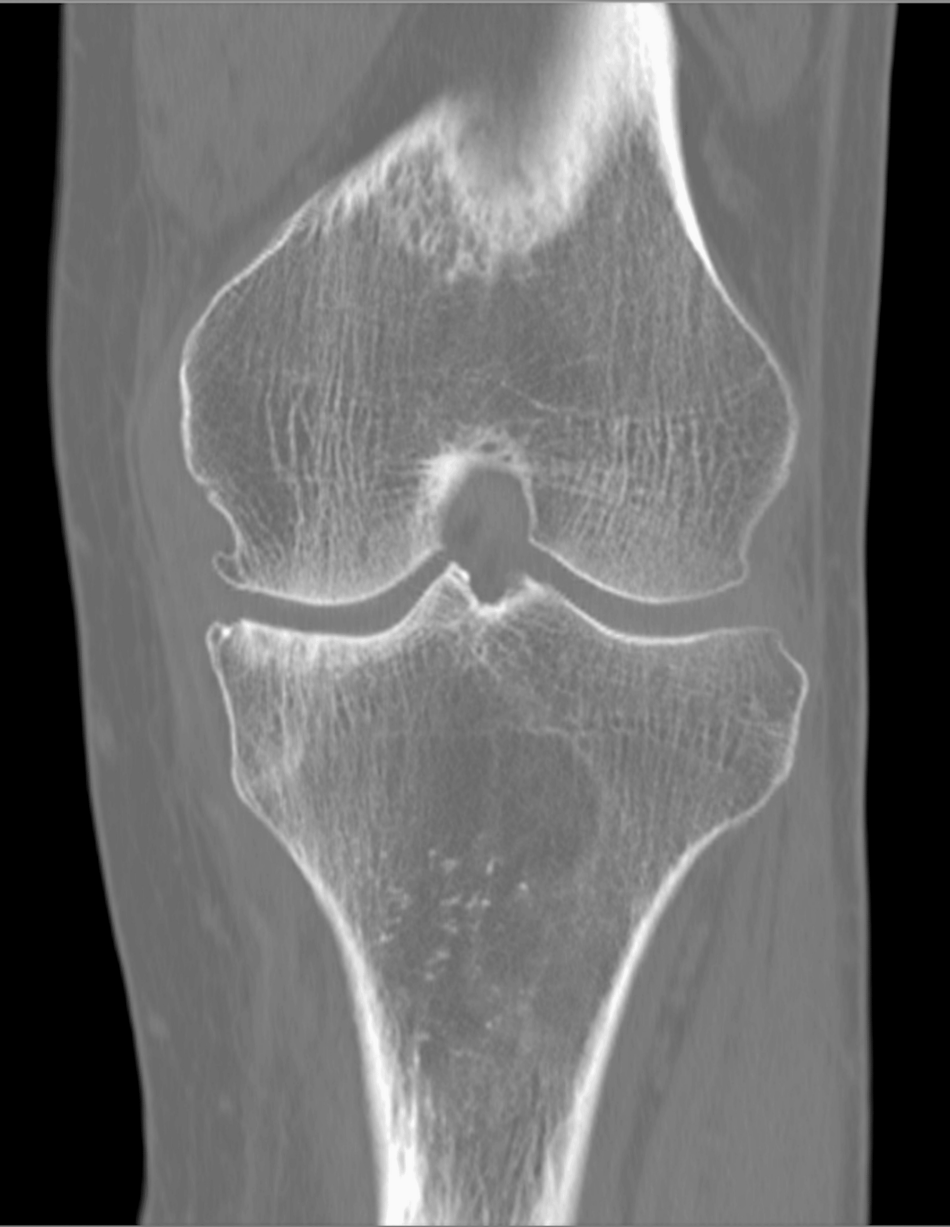
CT before the surgical treatment. CT showed osteophyte formation in the subchondral bone of the tibial plateau. Bone radiolucency was also observed at the metaphyseal end.

Computed tomography (CT) showed osteophyte formation in the subchondral bone of the tibial plateau. Radiolucency of the bone was observed at the metaphyseal end (Figure 2).

**Figure 3:**
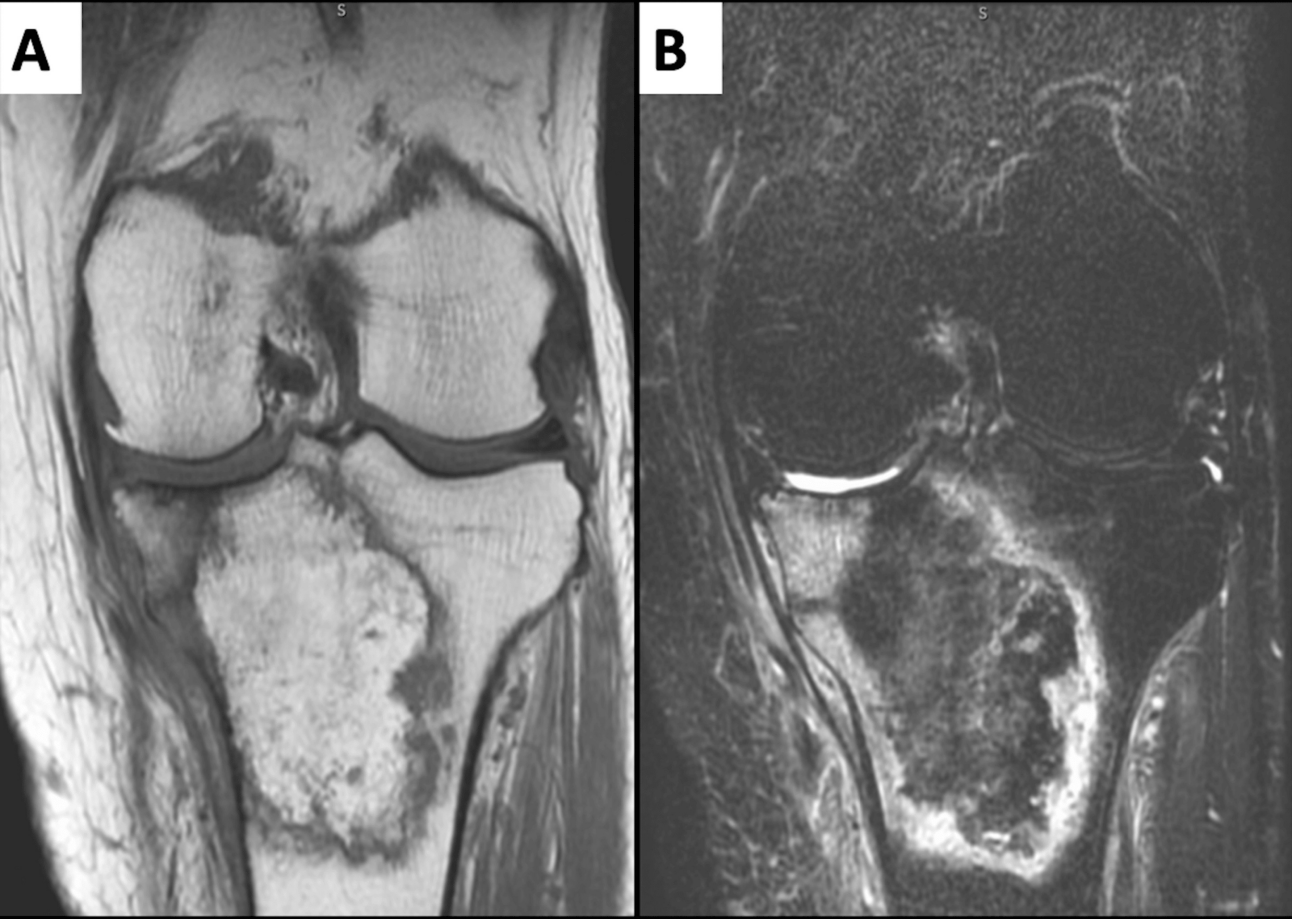
MRI before the surgical treatment. (A) MRI T1WI showed extensive high signal area in the proximal tibia. (B) MRI T2 fat sat showed a massive bone marrow region in the proximal tibia.

Magnetic resonance imaging (MRI) revealed a degenerative tear from the medial meniscus to the posterior horn and extensive abnormal signals from the tibial plateau to the diaphyseal end (Figure 3).

**Figure 4:**
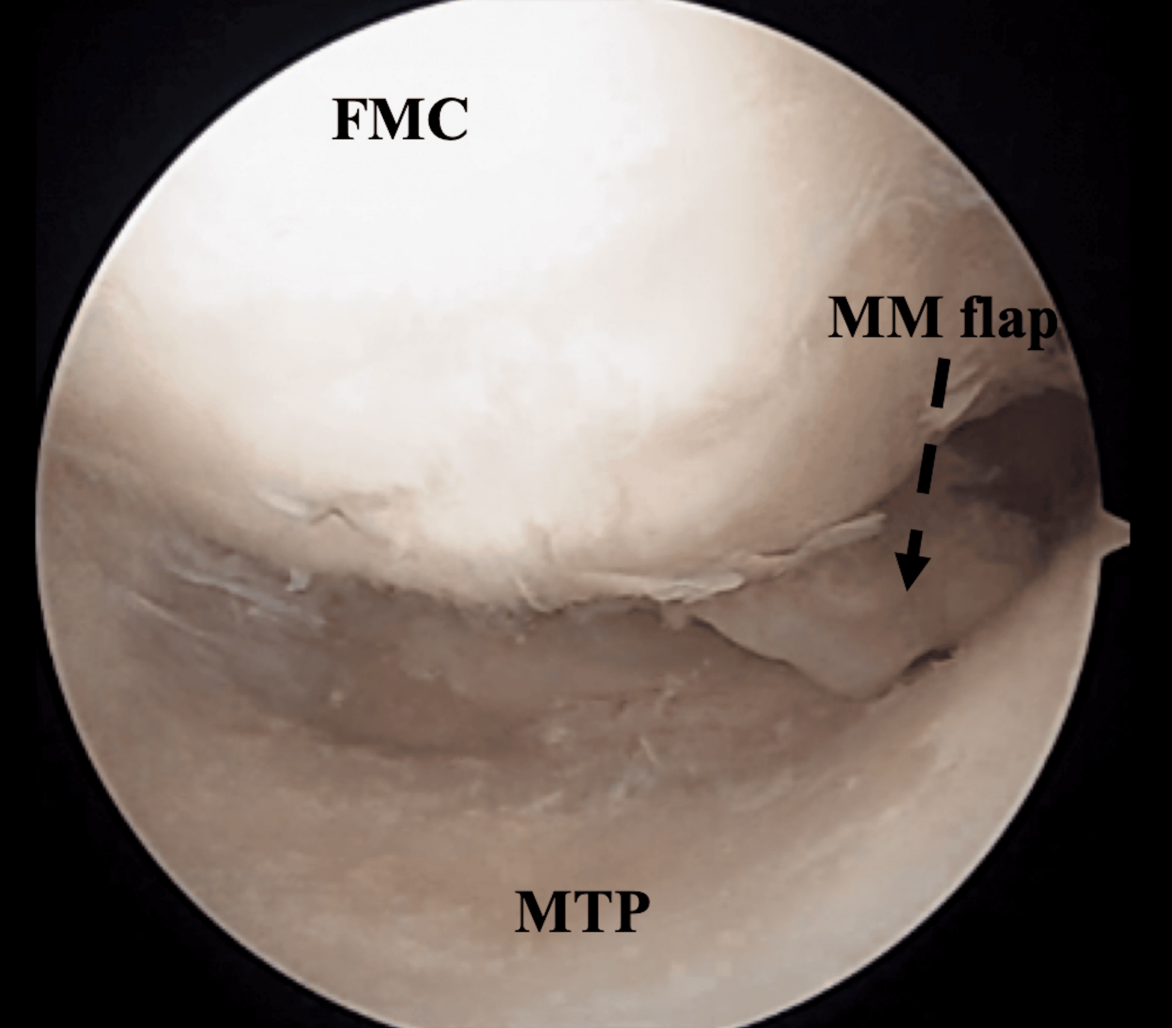
Arthroscopic assessment at the time of the surgical treatment. Cartilage region with ICRS Grade 2 at the femoral condyle and Grade 4 at the posterior end of the tibial condyle. A degenerative tear was observed in the posterior corner of the medial meniscus. FMC: Femoral medial condyle, MTP: Medial tibial plateau, MM: Medial meniscus.

Based on the above findings, idiopathic osteonecrosis of the tibial plateau was diagnosed and the patient was treated surgically. The surgical treatment consisted of the following interventions. Arthroscopy was performed prior to OWHTO. Arthroscopy revealed a cartilage region with International Cartilage Repair Society (ICRS) cartilage repair assessment grade 2 at the femoral condyle and 4 at the posterior end of the tibial condyle. A degenerative tear was observed at the posterior corner of the medial meniscus (Figure 4).

**Figure 5:**
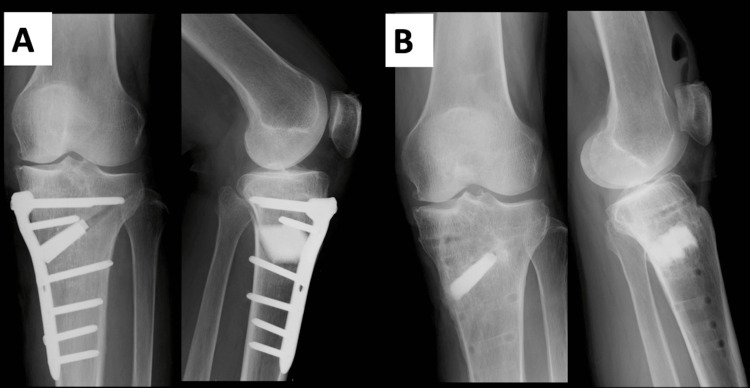
An X-ray after surgical treatment. (A) Biplanes OWHTO was performed with a TomoFix plate (DepuySynthes). β-Tricalcium phosphate was inserted into the osteotomy site. (B) Radiograph two years after surgery. The bone union has been achieved, and part of the artificial bone has been replaced with bone tissue.

A portion of the degenerative meniscus was excised, and the flap was sutured to the posterior articular capsule using an all-inside suture device (Stryker Air; Stryker, USA). Biplane OWHTO was performed using a TomoFix plate (DePuy Synthes, USA). Intraoperative alignment rods were used to correct the osteotomy, such that the line connecting the center of the femoral head and the center of the ankle joint passed through 63% of the mechanical axis of the knee. As a complete curettage of the necrotic area on the image would have resulted in a defect that was too large, a partial curettage was performed to freshen the border area with the lateral normal portion from the osteotomy area. The curettaged bone was subjected to pathological examination. β-Tricalcium phosphate (OSferion60, Olympus Terumo Biomaterials Corporation, Japan) was inserted into the osteotomy site (Figure 5).

**Figure 6:**
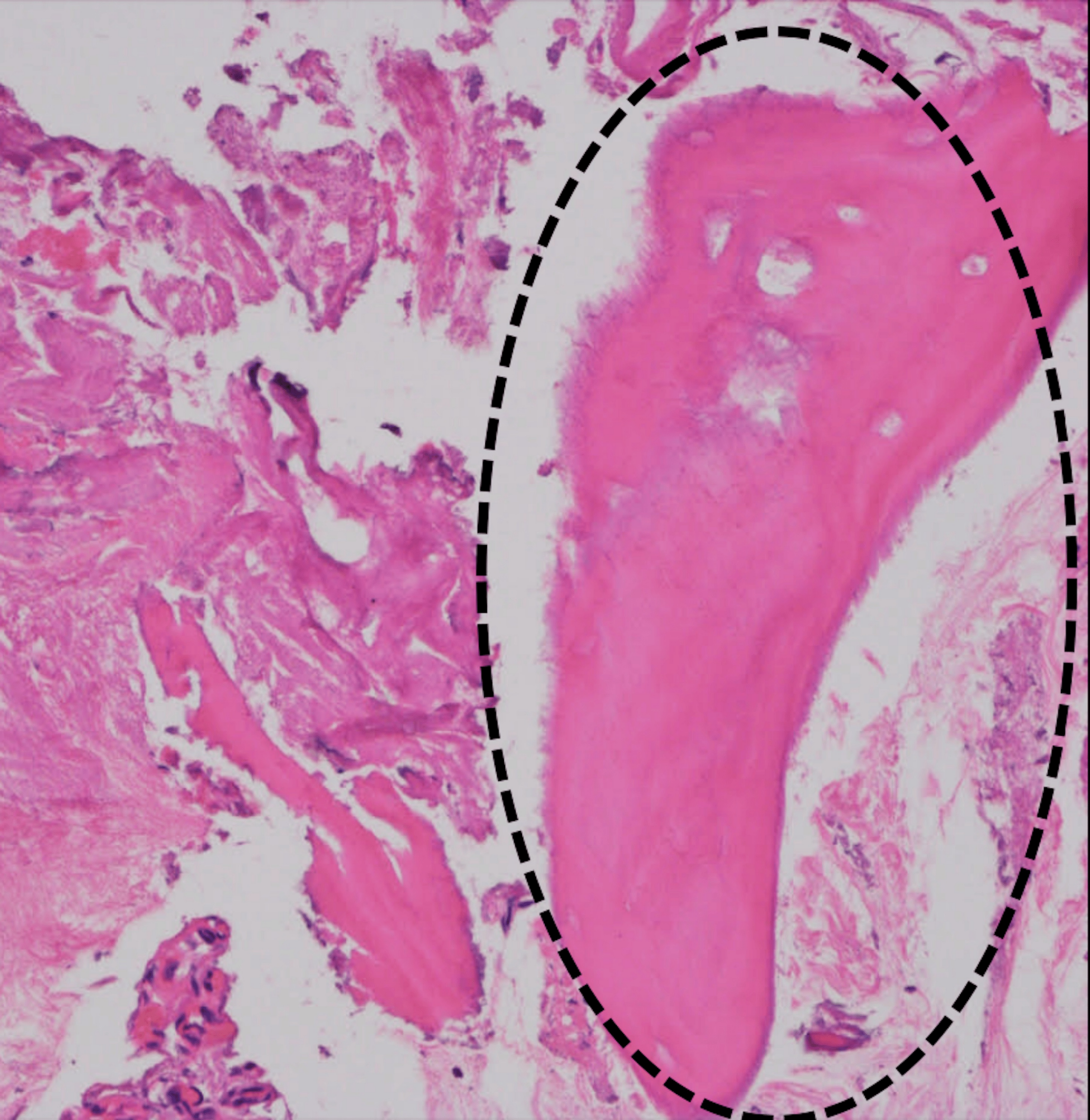
The pathological assessment. The pathological diagnosis was necrotic bone due to the absence of cells in the bony fossa (in the dotted line circle).

The patient was allowed half partial weight-bearing the day after surgery and whole weight-bearing one week later. The patient was discharged from the hospital to her home at five weeks postoperatively. The Japanese Knee Osteoarthritis Measure (JKOM) score value improved by 4 units three months postoperatively. The pathological diagnosis was necrotic bone owing to the absence of cells in the bony fossa (Figure 6).

**Figure 7:**
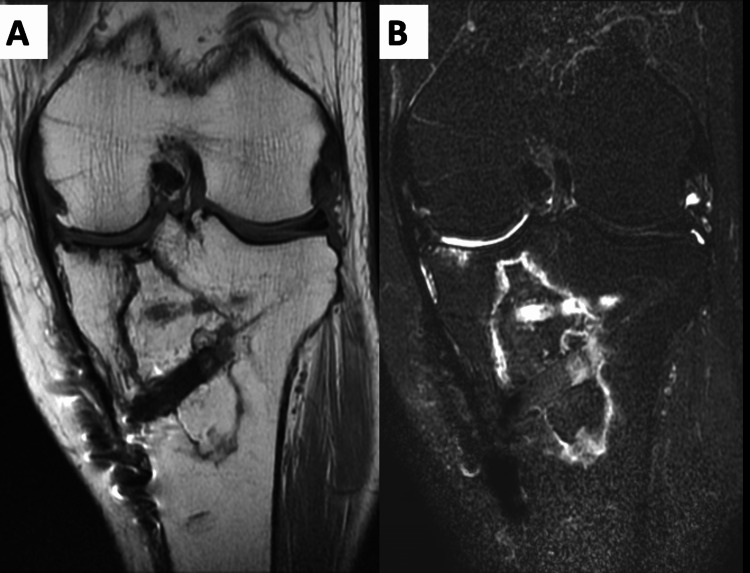
MRI two years after surgical treatment. (A) T1WI, (B) T2 fat sat. MRI showed a reduction in the extent of necrosis. In particular, the loading surface of the medial tibial condyle showed a prominent reduction in the extent of necrosis and bone remodeling.

The implant was removed 18 months after the surgery. MRI at the final follow-up revealed a reduction in the extent of necrosis. In particular, the loading surface of the medial tibial condyle showed a prominent reduction in the extent of necrosis and bone remodeling (Figure 7).

Clinical evaluations preoperatively and two years postoperatively at the final follow-up included an assessment of range of motion (ROM), JKOM score, and Knee Injury and Osteoarthritis Outcome Score (KOOS) values. Radiographs were obtained preoperatively and at the final follow-up (Table 1).

**Table 1:** Clinical and radiographic data of the preoperative evaluations and two years after operation. JKOM improved to 2 points two years after surgery and KOOS to 97.6 points. The %MA was also 60, and the lateralization of the weight-bearing axis was maintained. JKOM: Japanese Knee Osteoarthritis measure [12]; KOOS: Knee Injury and Osteoarthritis Outcome Score [13]; %MA: % Mechanical Axis; MPTA: Medial Proximal Tibial angle.

	Pre-operation	Two years after operation
Range of Motion		
Extension angle (°)	0	0
Flexion angle (°)	145	145
JKOM score Total	63	2
VAS	98	0
Pain	22	0
ADL	21	1
General	14	0
Health	6	1
KOOS Total	33.9	97.6
Pain	25	10
Symptom	50	96.4
ADL	44.1	100
Sports	15	90
QOL	6.2	93.7
Radiographic evaluation		
%MA	25	60
MPTA (°)	86	94

The KOOS score was 33.9 preoperatively and improved markedly to 97.6 at the final follow-up. The patient was pain-free, could engage in cleaning jobs, jog, and enjoyed yoga (Video 1).

**Video 1:** Jogging at two years postoperatively. The patient was pain-free and able to jog.

## Discussion

This study meticulously documented the clinical course of a patient with extensive spontaneous tibial plateau osteonecrosis who was treated with medial OWHTO. The patient's progression from initial pain presentation to imaging-confirmed extensive necrosis and subsequent postoperative recovery underscores the potential for significant symptomatic relief and functional improvement with OWHTO. Notably, the patient has shown significant pain relief and improved activity in a short period, as evidenced by a significant improvement in JKOM scores within the first three months postoperatively. Spontaneous osteonecrosis of the knee (SONK) is a condition that typically presents in patients over the age of 55 years with no associated risk factors for osteonecrosis, while those with secondary osteonecrosis are younger than 55 years and have associated risk factors such as a history of alcohol and corticosteroid use [1,14]. Secondary osteonecrosis typically involves multiple condyles. However, SONK usually involves only a single condyle or plateau, most commonly the medial femoral condyle. Spontaneous osteonecrosis of the medial tibial plateau is rare, accounting for only 2% of reported knee necrosis [1,15]. In 1982, Houpt et al. reported that tibial plateau osteonecrosis remains an incompletely understood disease due to the absence of a large patient series that has been systematically reviewed [3]. Therefore, limited information is available on the treatment and course of extensive SONK, especially the medial tibial plateau (MTP). The etiology of SONK is unclear, with two classic theories proposed: primary vascular insufficiency leading to bone infarction and trauma resulting in microfracture and subsequent osteonecrosis [5,6,16-18]. More recently, an association between subarticular stress responses or fractures, meniscal tears, and the resulting biomechanical changes has also been suggested [16-19]. Further, the concomitant occurrence of tibial plateau osteonecrosis and osteonecrosis of the adjacent femoral condyle supports the hypothesis that meniscal injuries play a pivotal role in the pathogenesis of this disease on both sides of the joint [20]. Previous reports have suggested that many SONK-like lesions detected on MRI are stress or insufficiency fractures with resultant necrosis [21,22]. Since symptoms can develop acutely with minor trauma, a predilection for the medial articular surface of the knee joint, and subchondral sclerosis within a few months in conjunction with symptomatic improvement, we recognize the relevance of reports showing that subchondral insufficiency fractures are the cause of necrosis [6,16,19]. Since osteonecrosis of the tibial plateau has been recognized as a cause of knee pain and progressive joint collapse, careful observation of symptoms and selection and timing of treatment are essential [3,6]. The present case also showed meniscal injury and medial shifting of the %mechanical axis, suggesting that changes in mechanical stress may have caused progressive necrosis. Based on arthroscopic findings, previous investigators have suggested that meniscal pathology may be the underlying etiology of knee osteonecrosis [20,23] and that meniscal dysfunction, including posterior root tears and meniscal extrusion, has been highly correlated with SONK at the femoral condyle [16,24]. A normal meniscus converts axial loading into tensile stress. This important mechanical property helps absorb the contact stresses applied to the knee [25-27]. There is growing recognition that tibial plateau osteonecrosis should be diagnosed at an early stage when appropriate management may prevent disease progression and provide a more favorable outcome for the patient [9,10]. A conservative treatment for tibial plateau osteonecrosis requires close follow-up. Patients with small stable lesions are treated conservatively with weight-bearing protection, activity modification, and anti-inflammatory medications [6,16,20,28-30]. If a presumptive diagnosis of stress reaction or progressive stress fracture is made, an appropriate treatment strategy is required to prevent bone collapse and severe late joint damage. The treatment for MTP osteonecrosis remains controversial. Conservative treatment with unloading, arthroscopic drilling, osteochondral grafting, HTO, unicondylar knee arthroplasty (UKA), and TKA have been reported. OWHTO is a well-established technique for the treatment of SONK of the femoral condyle and is expected to improve necrosis because of its effectiveness in reducing mechanical stress by improving alignment [7,11]. Therefore, we considered OWHTO to be the most reasonable treatment option for extensive osteonecrosis of the MTP. We observed a marked improvement in the extent of osteonecrosis in both symptoms and imaging evaluation. This was the strongest finding of this study. Satku et al. [31] reported the natural history of 21 cases of tibial osteonecrosis, with two cases experiencing acute extensive collapse within three months of onset and three cases rapidly progressing to severe osteoarthritis requiring TKA. Therefore, it is essential to perform HTO before extensive disease progression occurs. This study brings two new findings to the existing knowledge. First, pain did not appear until the extent of necrosis had expanded substantially, suggesting delayed detection in some patients. Second, it underscores the effectiveness of OWHTO in managing extensive spontaneous tibial plateau osteonecrosis, resulting in robust symptomatic relief and functional recovery. Our findings are consistent with the results of previous studies on the outcomes of osteonecrosis that support the use of surgical interventions for symptom management and functional improvement. While acknowledging the limitations inherent in single case studies, these findings provide valuable insights for clinicians considering surgical intervention for tibial plateau osteonecrosis. The results of this study were interpreted within the framework of osteonecrosis pathophysiology and surgical treatment efficacy. Delayed onset of pain after extensive necrosis may reflect the progressive nature of osteonecrosis. The effectiveness of OWHTO in this situation suggests that in addition to providing a viable surgical option, altering the mechanical axis can alleviate stress at the necrotic site, relieve symptoms, and promote functional recovery. This interpretation is consistent with the principles of biomechanics and osteonecrosis management and reinforces the clinical validity of OWHTO.

## Conclusions

In conclusion, this case report provided compelling evidence for the clinical utility of medial OWHTO in the treatment of spontaneous osteonecrosis of the extensive tibial plateau. This case report highlighted the potential for delayed symptom onset in patients with tibial plateau osteonecrosis and demonstrated the efficacy of OWHTO in providing significant symptom relief and functional improvement. Further studies are warranted to explore the broad applicability of OWHTO and to confirm its efficacy in a larger patient cohort.
